# Identification and Whole-Genome Sequencing of Four Enterovirus D68 Strains in Southern China in Late 2015

**DOI:** 10.1128/genomeA.01014-16

**Published:** 2016-09-22

**Authors:** Long Chen, Lei Shi, Hong Yang, Da-Yong Gu, Jun Meng, Yun-Qing Xu, Xiang-Jie Yao, Hai-Long Zhang, Jin-Quan Cheng, Han-Wu Ma, Ren-Li Zhang, Ya-Qing He

**Affiliations:** a Major Infectious Disease Control Key Laboratory and Shenzhen Public Service Platform of Pathogenic Microorganisms Repository, Department of Microbiology, Shenzhen Center for Disease Control and Prevention, Shenzhen, China; b Shenzhen International Travel Healthcare Center, Shenzhen, China

## Abstract

Four enterovirus D68 (EV-D68) strains from four children with influenza-like illness were identified in Shenzhen, southern China, in late 2015. Here, we announce the availability of these viral genomes in GenBank. The genomic sequences of these EV-D68 strains showed the closest phylogenetic relationship to strains from northern China.

## GENOME ANNOUNCEMENT

Enterovirus D68 (EV-D68), a member of the enterovirus D species of the family *Picornaviridae*, is a single stranded, positive-sense RNA virus with an ~7.3-kb genome (1, 2). Its genome comprises a 5′ untranslated region (5′-UTR), structural polypeptide P1, nonstructural polypeptides P2 and P3, and a 3′ untranslated region (3′-UTR). P1, P2, and P3 are cleaved into four structural proteins (VP1 to VP4), three nonstructural proteins (2A to 2C), and four nonstructural proteins (3A to 3D), respectively. EV-D68 was originally isolated from four American children with respiratory illness in 1962 (3). Since then, EV-D68 had not caused public concern until its outbreaks in the United States in the second half of 2014 (4–7). Subsequently, some retrospective and field epidemiological investigations on the prevalence of EV-D68 associated with respiratory infections were successively reported in different geographic regions (8–10). These data have demonstrated an increasing number of patients with EV-D68 infections and the wide spread of EV-D68 across the world in recent years.

Between 16 November 2015 and 16 December 2015, four EV-D68 strains from four children with influenza-like illness were identified by real-time reverse transcription PCR and sequencing of VP1 genes in Shenzhen, southern China (11). Next, two sets of primers pairs, EVD68-1F21 (TTAAAACAGCTCTGGGGTTGT)/EVD68-4570R27 (ATTTTGCATTAAATCATCCATAAGGAC) and EVD68-4394F26 (TCAAGTCCAAATCTCGCATTGAACCG)/EVD68-7340R27 (GTCCCCAAGTGACCAAAATTTACCTCT), were designed to amplify two overlapping fragments encompassing the complete genome of EV-D68. Amplified DNA products were sequenced by TaKaRa using a primer-walking method. The two overlapping fragments were assembled into a complete genome using Lasergene version 7.1. The assembled genome sequences were examined using BioEdit version 7.2.5 before submission to GenBank.

The complete genome sequences of the four EV-D68 strains from the study were composed of 7,293 nucleotides (nt), excluding the poly(A) tail. The 5′-UTR contains 699 nt, followed by an open reading frame encoding the structural protein P1 (2,583 nt), the nonstructural proteins P2 (1,728 nt) and P3 (2,253 nt), and the 3′-UTR (27 nt). The base compositions of the full genomes of the four EV-D68 strains are 31.58 to 31.67% A, 20.35 to 20.47% C, 21.34 to 21.42% G, and 26.53 to 26.64% U. The complete nucleotide sequences and complete amino acid sequences (2,188 aa) of the four EV-D68 strains showed 0 to 2.9% and 0 to 0.8% differences to each other, respectively. No sequence insertion or deletion was observed in the complete genome region of the four EV-D68 strains compared to the closest known strains. Genome-wide sequence analysis indicated that these four EV-D68 strains belonged to the latest clade comprising some sporadic strains from China.

Recent EV-D68 strains have evolved worldwide into distinct clades, which shows the trend of further geographical spread (6, 12–18). Since there is no epidemiological data on EV-D68 infections in southern China at present, this study urges us to investigate the prevalence of EV-D68 as quickly as possible and strengthen surveillance of EV-D68 to prevent its outbreak.

### Accession number(s).

The complete genome sequences of four EV-D68 strains from the present study have been deposited in GenBank under the accession numbers KU982558 to KU982561.
